# Ginsenoside-Enriched *Panax ginseng* Sprouts Cultivated from Aquaponic System with a Novel Nutrient Solution Regulate LPS-Induced Inflammatory Cytokines and UVB-Induced Photoaging Responses via MAPK/AP-1 Signaling Pathways

**DOI:** 10.3390/plants14111712

**Published:** 2025-06-04

**Authors:** Jeong-Ho Kim, Kyung-Wuk Park, Beom-Gyun Jeong, Jun-Ki Park, Ho-Yeol Jang, Yun-Seo Oh, Jin-Yeong Choi, Kyung-Yun Kang

**Affiliations:** R&D Team, Suncheon Research Center for Bio Health Care, Suncheon-si 57962, Republic of Korea; kimjeoho90@gmail.com (J.-H.K.); uk988446@sbrc.kr (K.-W.P.); fusionchef@sbrc.kr (B.-G.J.); nada3663@naver.com (J.-K.P.); yeol2686@naver.com (H.-Y.J.); yun-seo@sbrc.kr (Y.-S.O.); jychoi@sbrc.kr (J.-Y.C.)

**Keywords:** *Panax ginseng* sprouts, kelp fermentates, crude saponin fractions, ginsenosides, antioxidant, anti-inflammatory, anti-photoaging

## Abstract

*Panax ginseng* sprouts (GSs) have attracted attention as functional resources due to their short cultivation time and enriched ginsenoside content. This study aimed to evaluate the bioactivities of GSs cultivated using kelp fermentates (KF) as a nutrient solution under a smart-farming system. Ginsenoside-enriched extract (FGE), its water-soluble saponin fraction (WFGE), and 70% ethanol-soluble saponin fraction (EFGE) were analyzed for phytochemical contents and biological activities. The EFGE exhibited the highest levels of eight major ginsenosides, including Rg1, Rb1, Rc, Rg2, Rb2, Rd, Rf, and F2. Total phenolic and flavonoid contents were significantly higher in KF-treated ginseng and their crude saponin fractions, with EFGE showing the highest values. WFGE and EFGE indicated strong antioxidant activity through ABTS radical scavenging assays. In LPS-stimulated RAW264.7 macrophages, all extracts significantly inhibited nitric oxide production and downregulated IL-1β, IL-6, iNOS, and COX-2 expression. Moreover, UVB-irradiated human fibroblasts (Hs68) treated with KF-derived fractions showed increased cell viability, enhanced procollagen synthesis, and reduced MMP-1 and MMP-3 expression. These effects were associated with suppression of MAPK/AP-1 signaling. In conclusion, GSs cultivated with KF exhibit notable antioxidant, anti-inflammatory, and anti-photoaging activities, suggesting their potential as natural ingredients for skin health applications.

## 1. Introduction

The skin, as the body’s largest organ, consists of the epidermis, dermis, and hypodermis, covering the external surface and providing protection against various external stimuli such as toxic chemicals, infections, and ultraviolet (UV) irradiation [1]. Repeated excessive exposure to solar UV, particularly UVB, can lead to photoaging, resulting in dryness, wrinkles, and loss of skin elasticity [2]. UV irradiation triggers inflammatory responses by upregulating cytokines like tumor necrosis factor-α (TNF-α), interleukin (IL)-1β and IL-6, and contributes to extracellular matrix (ECM) degradation, which constitutes the structure of skin tissues through increased expression of matrix metalloproteinases (MMPs) [3,4]. Many researchers have shown that the activator protein (AP)-1 signaling pathway, influenced by activated mitogen-activated protein kinases (MAPKs) such as p38, extracellular response kinase (ERK), and c-Jun N-terminal kinase (JNK), plays a crucial role in regulating UVB-induced photoaging [5,6]. Also, it is known that UV irradiation stimulates the immunosuppression responses to cellular senescence with increased inflammation levels within the body [7]. Based on previous studies, we concluded that inflammatory response assessment is closely related to photoaging responses.

*Panax ginseng* Meyer, commonly referred to as ginseng, has been used as a folk medicine in Asian countries for over 2000 years [8]. Extensive research has examined the phytochemicals present in ginseng, including triterpenoid saponins, phenolic compounds, and vitamins [9,10,11]. Among these phytochemicals, triterpenoid saponins, known as ginsenosides, are the primary biologically active compounds, offering anti-inflammatory, anti-melanogenic, and anti-photoaging benefits for damaged skin [12,13,14]. Despite its high cost and lengthy cultivation period, ginseng is a valuable resource in the nutraceutical and cosmetic industries [15]. To improve cultivation efficiency, effectiveness, and productivity, smart-farming systems have been proposed as an alternative for growing ginseng sprouts, managing growth conditions such as temperature, humidity, and lighting [16]. Consequently, both researchers and the agricultural industry have shown significant interest in ginseng sprouts (GS) due to their shorter cultivation period and high accumulation of biologically active compounds [15]. Smart farming has gained attention as a sustainable strategy to enhance the efficiency of plant cultivation; however, the widespread use of synthetic chemical-based nutrient solutions in hydroponic systems raises concerns about environmental impact and residue accumulation [17]. Consequently, alternative nutrient sources derived from natural resources such as seaweeds including *Sargassum polycystum* and *Nizamuddinia zanardinii* are gaining traction due to their biostimulant properties and ecological safety [18,19]. In this context, our previous study demonstrated that one-year-old GSs cultivated using kelp (*Laminaria japonica*) fermentates (KF) as a nutrient solution under a smart-farming system significantly accumulated levels of key ginsenosides including Rg1, Re, Rb1, Rc, Rg2, Rb2, and Rd [20].

Despite increasing interest in preventing inflammation and UV-induced skin aging, the biological effects of GSs cultivated using KF as a nutrient solution have not yet been thoroughly investigated. Therefore, the present study aimed to evaluate the anti-inflammatory and anti-photoaging effects of ginsenoside-enriched GSs grown hydroponically with KF in lipopolysaccharide (LPS)-stimulated macrophages and UVB-irradiated human fibroblasts in vitro.

## 2. Results and Discussion

### 2.1. Bioactive Compounds Contents in Ginseng Sprouts Cultivated with Kelp Fermentates and Its Antioxidant Activities

**Figure 1:**
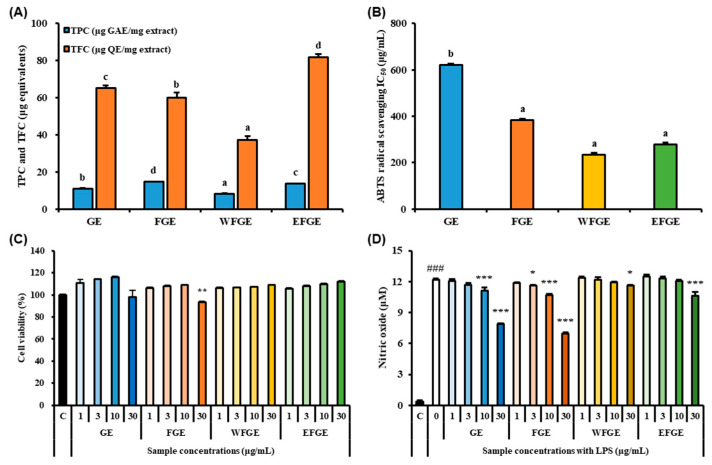
Total phenolic contents (TPC) and total flavonoid contents (TFC) (**A**), IC50 values of ABTS radical scavenging activity (**B**), cell viability (**C**) and nitric oxide (NO) production (**D**) of ginseng extracts (GE), the extract of kelp fermentates−treated ginseng (FGE), the water fraction of crude saponin extract from FGE (WFGE), and the 70% ethanol fraction of crude saponin extract from FGE (EFGE). RAW264.7 cells were used to determine cytotoxicity and NO production. Data values are expressed as mean as S.D. of triplicate determinations. Different letters on the bar (**A**,**B**) show the differences in Duncan’s multiple range tests (*p* < 0.05). Significant differences were compared with control at * *p* < 0.05, ** *p* < 0.01, and *** *p* < 0.001 and with LPS group at ^###^ *p* < 0.001 by one−way analysis of variance and Tukey’s multiple comparison (**C**,**D**).

Ginseng contains assorted phenolic and flavonoid compounds such as caffeic acid, *p*-coumaric acid, vanillic acid, protocatechuic acid, quercetin, and epigallocatechin gallate which are known as biologically active compounds [21,22]. Also, previous studies have reported that *Panax ginseng*, which contains high levels of representative ginsenosides such as Rg1, Rb1, and Rc, exhibits significant free radical scavenging and Fe^2^⁺-chelating activities, contributing to its antioxidant capacity [23,24]. The evaluation of total phenolic contents (TPC) and total flavonoid contents (TFC) was carried out in ginseng extracts (GE) and their fractions from an extract of GS cultivated with KF as a nutrient solution (Figure 1A). The extract of KF-treated GS (FGE) (14.77 ± 0.14 μg GAE/mg) showed a significant elevation of TPC compared with GE (11.09 ± 0.24 μg GAE/mg). The water fraction of crude saponins extract from FGE (WFGE, 8.23 ± 0.24 μg GAE/mg) and the 70% ethanol fraction of crude saponins extract from FGE (EFGE, 13.61 ± 0.11 μg GAE/mg) obtained from the crude saponin extract of FGE exhibited significant differences, likely due to the ethanol-based fractionation process, which separates phytochemical constituents based on their polarity. However, the total flavonoid content (TFC) of GE (65.22 ± 1.43 µg QE/mg) was significantly higher than that of FGE (59.92 ± 2.86 µg QE/mg). In the crude saponin fractions, WFGE (37.18 ± 2.16 µg QE/mg) and EFGE (81.73 ± 1.87 µg QE/mg) showed a similar trend to the TPC values. The radical scavenging activities of ginseng sprouts were determined by the 2,2′-azino-bis (3-ethylbenzothiazoline-6-sulfonic acid) (ABTS) radical scavenging assay and IC_50_ values were calculated, as presented in Figure 1B. The IC_50_ values of FGE (383.71 ± 1.16 μg/mL), WFGE (234.96 ± 1.53 μg/mL), and EFGE (279.25 ± 1.47 μg/mL) were significantly lower than GE (622.31 ± 1.09 μg/mL). Although the IC_50_ values of all samples were higher than that of the positive control (ascorbic acid, <10 μg/mL), FGE, WFGE, and EFGE exhibited notable radical scavenging activity. Cytotoxicity of GE, FGE, WFGE, and EFGE in RAW264.7 cells was assessed using the Cell Counting Kit-8 (CCK-8) assay (Figure 1C). The cell viabilities of RAW264.7 cells treated with GE, FGE, WFGE, and EFGE remained above 93% at concentrations of 1, 3, 10, and 30 µg/mL. However, FGE showed a slight but significant cytotoxicity (93.08 ± 1.21%) at the highest dose. To evaluate nitric oxide (NO) inhibition, RAW264.7 cells were stimulated with 1 µg/mL LPS prior to treatment with GE, FGE, WFGE, or EFGE. As shown in Figure 1D, LPS stimulation markedly increased NO levels compared to the control group. However, treatment with GE, FGE, WFGE, or EFGE significantly suppressed NO production in a dose-dependent manner. Free radicals such as hydroxyl radical, superoxide anion, and lipid peroxyl are molecules or atoms carrying one or more unpaired electrons and existing independently, thus they are easily converted to reactive oxygen species (ROS) radical derivatives, such as singlet oxygen and hydrogen peroxide, which cause numerous diseases including diabetes, cancer, inflammation, ischemia, and anemia [25,26]. Hence, many researchers have reported that the biologically active compounds exert antioxidant capacities and anti-inflammatory activities [27,28]. *Lactobacillus*-fermented ginseng extract significantly showed increased TPC and reduced TFC after the fermentation process affected antioxidant capacity and inhibition of NO production [29]. Therefore, this result suggests that FGE and its derived crude saponins fractions (WFGE and EFGE) include abundant biologically active compounds, and they might exert antioxidant capacities and anti-inflammation activity.

### 2.2. Effect of Ginseng Sprouts Cultivated with Kelp Fermentates on LPS-Induced Inflammatory Responses in Macrophages

**Figure 2:**
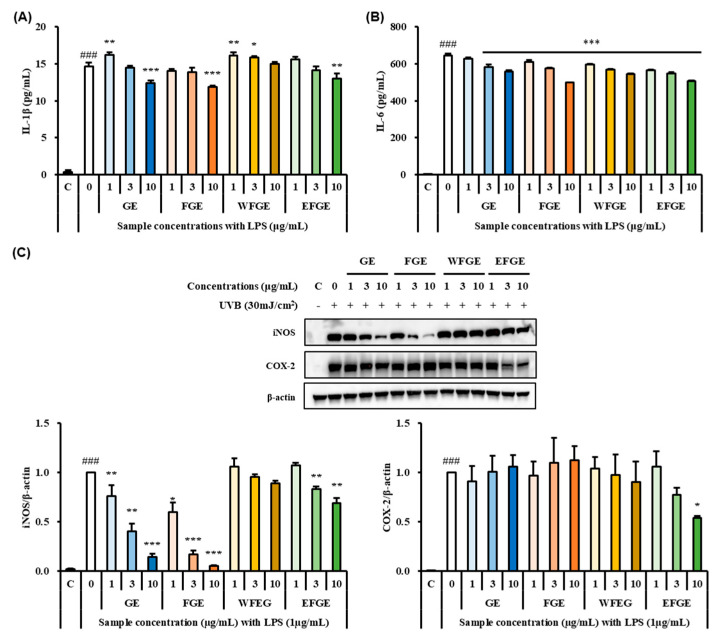
Effects of ginseng extracts (GE), the extract of kelp fermentates−treated ginseng (FGE), the water fraction of crude saponin extract from FGE (WFGE) and the 70% ethanol fraction of crude saponin extract from FGE (EFGE) on interleukin (IL)−1β (**A**) and IL−6 (**B**), and expressions of iNOS and COX−2 (**C**) in LPS−stimulated RAW 264.7 cells. Data values are expressed as mean as S.D. of triplicate determinations. Significant differences were compared with control at * *p* < 0.05, ** *p* < 0.01, and *** *p* < 0.001 and with LPS group at ^###^ *p* < 0.001 by one−way analysis of variance and Tukey’s multiple comparison.

The treatment of LPS upregulates the secretion of pro-inflammatory cytokines such as interleukin(IL)-1β and IL-6 in macrophages, and also the stimulation by LPS induces the activation of inflammatory mediators such as NO, inducible nitric oxide synthase (iNOS), and cyclooxygenase-2 (COX-2) which contribute to several physiological and pathological diseases [30]. Hence, many researchers have targeted the inhibition of pro-inflammatory cytokines and related enzymes to probe anti-inflammation activity of natural products [31]. To assess the regulation of inflammatory responses of GE, FGE, WFGE, and EFGE, the secretion of pro-inflammatory cytokines serving as IL-1β and IL-6, and the expressions of iNOS and COX-2 in LPS-stimulated macrophages, were evaluated by ELISA kits and Western blot assay. As shown in Figure 2A, the LPS group with non-treated samples showed significant elevation of IL-1β compared with control. However, GE, FGE, and EFGE significantly reduced the levels of IL-1β at the concentration of 10 μg/mL in LPS-treated control. The levels of IL-6 were decreased by a dose-dependent manner with strong significances compared with the LPS group (*p* < 0.001) (Figure 2B). To determine whether or not GE, FGE, WFGE, and EFGE inhibit the expression levels of iNOS and COX-2, a Western blot assay was carried out (Figure 2C). The expressions of iNOS and COX-2 were extremely increased in the LPS-treated group without samples. However, the treatment of GE, FGE, and EFGE significantly inhibited the protein expression of iNOS in LPS-stimulated macrophages with a dose-dependent manner. Then, EFGE showed a significantly down-regulated expression of COX-2 at a concentration of 10 μg/mL. Murakami et al. [31] reported that the increased secretion of pro-inflammatory cytokines may influence the expression of iNOS and COX-2. Yu et al. [32] reported that the crude saponins extract of the tissue cultured ginseng root ameliorated inflammatory responses such as NO production and the inhibition of pro-inflammatory cytokines (tumor necrosis factor-α (TNF-α) and IL-1β) and iNOS via NF-κB/MAPK signaling pathways. In addition, Gao et al. [33] reported that ginsenoside Rb1 exerted anti-inflammatory effects with the down-regulation of inflammatory mediators such as TNF-α, IL-6, IL-1β, iNOS, and COX-2 in LPS-stimulated murine macrophage RAW 264.7 cells via NF-κB/MAPK signaling pathways. Hence, this result suggests that the GE, FGE, and EFGE might exert anti-inflammation activity through the regulation of inflammatory responses.

### 2.3. Protective Effect of Ginseng Sprouts Cultivated with Kelp Fermentates on Extracellular Matrix and MMPs in UVB-Irradiated Fibroblasts

**Figure 3:**
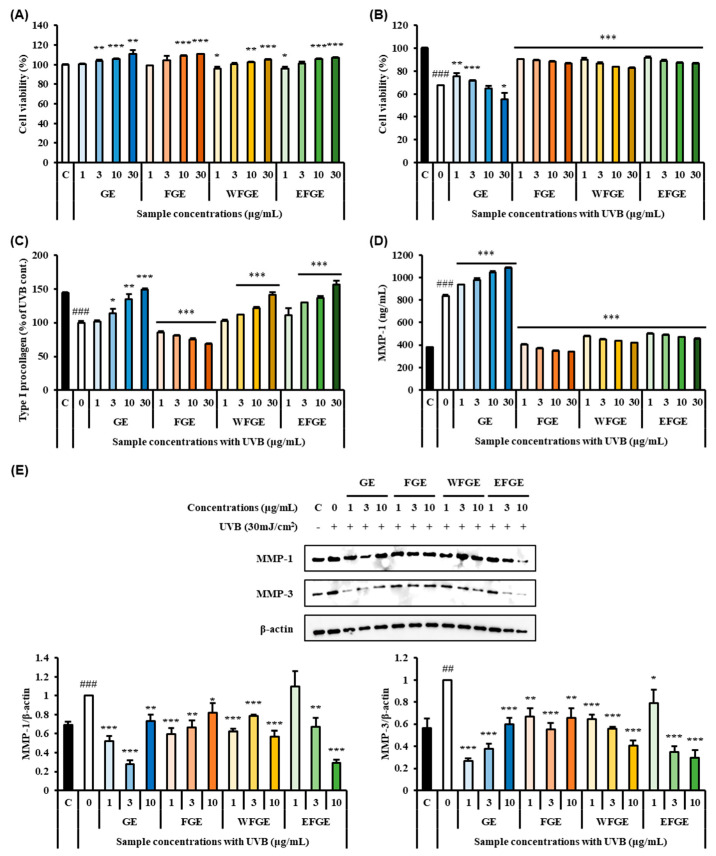
Effects of ginseng extracts (GE), the extract of kelp fermentates−treated ginseng (FGE), the water fraction of crude saponin extract from FGE (WFGE) and the 70% ethanol fraction of crude saponin extract from FGE (EFGE) on cell viability (**A**,**B**), procollagen (**C**) and MMP−1 (**D**), and the expressions of MMP−1 and MMP−3 (**E**) in UVB−irradiated Hs68 cells. Data values are expressed as mean as S.D. of triplicate determinations. Significant differences were compared with control at * *p* < 0.05, ** *p* < 0.01, and *** *p* < 0.001, and with the UVB group at ^##^ *p* < 0.01 and ^###^ *p* < 0.001 by one−way analysis of variance and Tukey’s multiple comparison.

UVB irradiation induces inflammation of skin via increased secretion of pro-inflammatory cytokines and leads to oxidative stress, which plays an important role in activating keratinocytes and macrophages to produce ROS [34]. In chronically inflamed tissues, pro-inflammatory cytokines and MMPs are released, and they might cause the ECM molecules to degrade [35]. Therefore, we evaluated the anti-photoaging effects of GE, FGE, WFGE, and EFGE on ECM and MMPs as photoaging biomarkers in 30 mJ/cm^2^ of UVB-irradiated human fibroblast Hs68 cells by ELISA kits and Western blot assay. In Figure 3A, all samples did not inhibit the proliferation of human fibroblast Hs68 cells, which means no cytotoxicity of GE, FGE, WFGE, and EFGE. When UVB-irradiated, the cell viability of the UVB group without sample treatment showed a significant inhibition (Figure 3B). Although GE showed a significant elevation of cell viability at 1 μg/mL and 3 μg/mL concentrations, FGE, WFGE, and EFGE markedly exerted greater protection shown over 90% of cell viabilities (*p* < 0.001) on UVB-damaged Hs68 cells from 1 μg/mL to 30 μg/mL concentrations. The production of procollagen was significantly decreased by 44.03 ± 1.45% after UVB exposure; however, GE, WFGE, and EFGE at a concentration of 30 μg/mL significantly resulted in 149.03 ± 1.22%, 140.81 ± 4.36%, and 155.97 ± 5.85%, respectively, which is significant considering the recovery from UVB-induced damages (Figure 3C). In Figure 3D, MMP-1, another biomarker of skin photoaging, strongly increased in the UVB-irradiated control group (836.29 ± 9.15 ng/mL) after UVB exposure, which compared with the control group (378.53 ± 3.48 ng/mL). However, the treatment of FGE, WFGE, and EFGE markedly inhibited the level of MMP-1, similar to the control group, with great significances (compared to the UVB control group, *p* < 0.001), though GE increased MMP-1 contents. The protein expression of MMP-1 and MMP-3 significantly increased under UVB exposure compared with the control group (Figure 3E). However, the treatment of all samples induced significant inhibition of MMP-1 and MMP-3. Thus, this result indicates that the extracts of ginseng sprouts and its crude saponins fractions effectively protected UVB-induced ECM damages in Hs68 cells. Similarly, Mariné-Casadó et al. [36] investigated the anti-photoaging activity of pomegranate extract on UV-irradiated Hs68 cells. In the study, the administration of the extract ameliorated skin health-related parameters such as procollagen, hyaluronic acid, and MMP-1. Park et al. [37] reported that carnosic acid isolated from rosemary modulated UV-induced responses related to ECM such as the expression of MMP-1, MMP-3, and MMP-9, and cellular mechanisms in human fibroblasts Hs68 cells via MAPK/AP-1 signaling pathways. Therefore, this result indicates that FGE and its derived crude saponin fractions protect UVB-damaged procollagen and induce down-regulation of MMPs in Hs68 cells.

### 2.4. Regulation of Ginseng Sprouts Cultivated with Kelp Fermentates on MAPK/AP-1 Signaling Pathways in UVB-Irradiated Hs68 Cells

**Figure 4:**
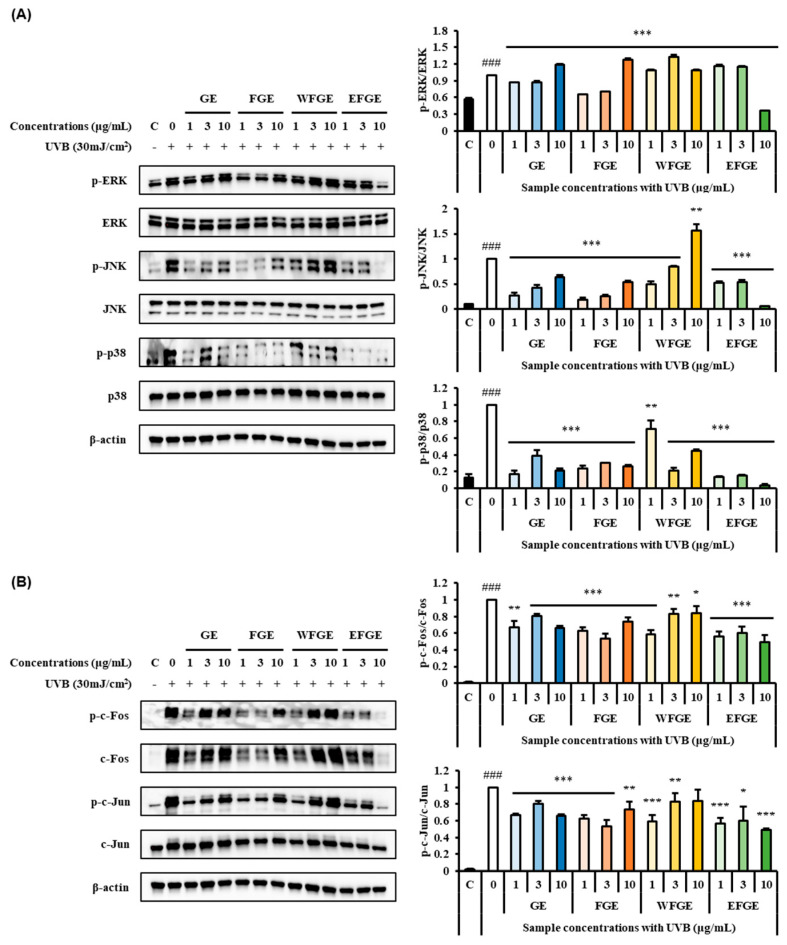
Effects of ginseng extracts (GE), the extract of kelp fermentates−treated ginseng (FGE), the water fraction of crude saponin extract from FGE (WFGE) and the 70% ethanol fraction of crude saponin extract from FGE (EFGE) on expressions of mitogen−activated protein kinases (**A**) and activator protein−1 (**B**) signaling pathways in UVB−irradiated Hs68 cells. Data values are expressed as mean as S.D. of triplicate determinations. Significant differences were compared with control at * *p* < 0.05, ** *p* < 0.01, and *** *p* < 0.001 and with UVB group at ^###^ *p* < 0.001 by one−way analysis of variance and Tukey’s multiple comparison.

The regulation of MAPK/AP-1 signaling pathways is closely related to MMPs on photoaging responses regulating MMPs expression, cell proliferation, and differentiation in human skin [38]. The MAPK transfers extracellular signals to the nucleus, resulting from activating transcription factors and leading target gene expression [39]. The AP-1, a MAPK downstream activator, is a transcription factor that triggers gene transcription of MMPs [40]. To explore the expression of phosphorylated forms of MAPK/AP-1 signaling pathways by the administration of GE, FGE, WFGE, and EFGE in UV-exposed Hs68 cells, Western blot assay was carried out. As shown in Figure 4A, UVB of 30 mJ/cm^2^ irradiation significantly induced the expression of p-ERK, p-JNK, and p-p38 compared to the control group. In the phosphorylation of ERK, GE and FGE of 1 μg/mL and 3 μg/mL and EFGE of 10 μg/mL were significantly reduced. In the phosphorylation of JNK, all groups showed a marked decrease in expression, except WFGE at a dose of 10 μg/mL. In the phosphorylation of p38, the expressions of all groups were significantly down-regulated. Particularly, EFGE effectively induced the inhibition of the MAPKs family. This result indicates that the extracts of ginseng sprouts and its crude saponin fractions regulate MAPK activation in UV-irradiated Hs68 cells. As shown in Figure 4B, the phosphorylated c-Fos and c-Jun were increased through the exposure of UVB compared to the control group. However, as treated samples, the expressions of all groups were significantly attenuated in the phosphorylation of c-Fos and c-Jun. This result reveals that the regulation of the expression of AP-1 resulted in the phosphorylation of the MAPKs family. Previous studies have reported that UVB-induced damages are recovered MAPK/AP-1 signaling pathways. The extract of *Eisenia bicyclis* Setchell improved the production of procollagen and inhibited MMP-1 production via suppressing MAPK/AP-1 signaling by enhancing the expression of tissue inhibitors of metallopeoteinases in UVB-irradiated Hs68 cells [38]. Ginseng seed extract displayed anti-photoaging activity on UVB-induced damaged Hs68 cells, resulting in the regulation of ROS, MMP-1, MMP-3, and collagen degradation via the inhibition of the phosphorylation of MAPK/AP-1 signaling pathways [41]. Also, the extract of cultivated ginseng root ameliorated the inhibition of collagen degradation and the elevation of MMPs via the suppression of UVB-induced activation of MAPK/AP-1 and NF-κB signaling pathways in human dermal fibroblasts [42].

### 2.5. Ginsenoside Contents in Ginseng Sprout Extract and Its Crude Saponins Fractions Cultivated with Kelp Fermentates

**Figure A1:**
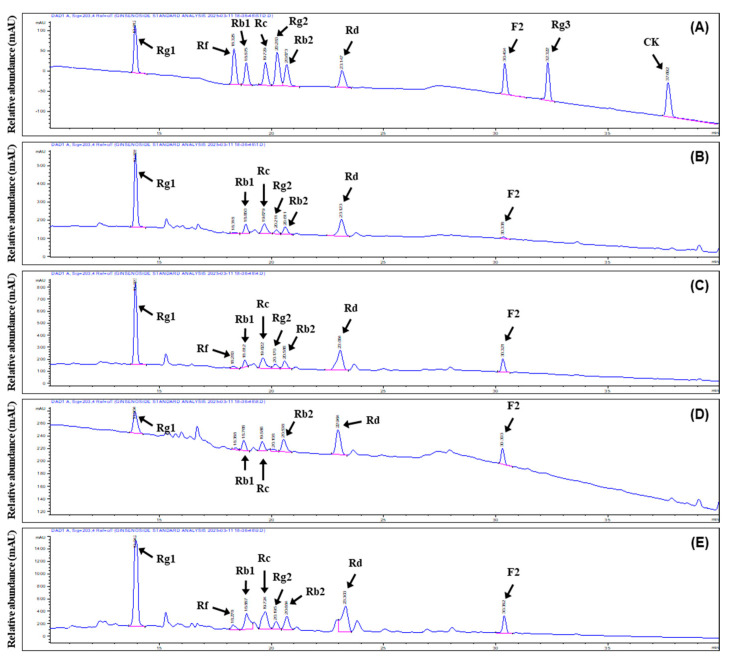
Chromatogram of ginsenosides and compound K (CK) in ginseng extracts (GE), the extract of kelp fermentates−treated ginseng (FGE), the water fraction of crude saponin extract from FGE (WFGE), and the 70% ethanol fraction of crude saponin extract from FGE (EFGE) as analyzed by high performance liquid chromatography-evaporative light scattering detector/mass spectrometry. (**A**) Standards of nine ginsenosides (Rg1, Rf, Rb1, Rc, Rg2, Rb2, Rd, F2, and Rg3) and CK; (**B**) GE; (**C**) FGE; (**D**) WFGE; (**E**) EFGE.

Ginsenosides, a class of triterpenoid saponins representative to the *Panax* species, comprise over 120 known compounds and are recognized for their diverse pharmacological activities [43]. Due to their significant functional potential, ginsenosides have been extensively studied in various biological and functional properties. Processing methods such as steaming and enzymatic treatment can modulate the natural content of ginsenosides in ginseng, often enhancing their concentrations [20]. In this study, we used high-performance liquid chromatography-evaporative light scattering detector/mass spectrometry (HPLC-ELSD/MS) to quantify nine ginsenosides (Rg1, Rf, Rb1, Rc, Rg2, Rb2, Rd, F2, Rg3, and compound K (CK)) in ginseng extracts and their saponin-enriched fractions. Ginsenosides Rg1, Rb1, Rc, Rb2, Rd, and F2 were detected across all sample groups. However, Rg3 and CK were not observed in any of the analyzed extracts (Figure A1). Notably, ginsenoside Rf was absent in extracts derived from ginseng sprouts cultivated with tap water, but was detected in those grown with KF solution and EFGE. Method validation was conducted using several analytical performance parameters including limits of detection (LOD) and quantification (LOQ). As summarized in Table A1, calibration curves for the ten analytes exhibited excellent linearity, with correlation coefficients (r^2^) exceeding 0.9993. LOD values ranged from 1.13 to 6.83, while LOQ values ranged from 11.34 to 68.22.

The quantified concentrations of nine selected ginsenosides and CK in GE, FGE, WFGE, and EFGE are summarized in Table 1. When compared to GE cultivated with tap water, the FGE cultivated with KF as a nutrient solution showed significantly elevated levels of ginsenosides Rg1, Rf, Rb1, Rc, Rg2, Rb2, Rd, and F2. Among the crude saponin fractions isolated from FGE, the 70% ethanol-soluble fraction (EFGE) contained the highest concentrations of all eight detectable ginsenosides as observed in the chromatographic analysis (Rg1: 433.39 ± 32.07 μg/g, Rf: 13.90 ± 2.59 μg/g, Rb1: 68.48 ± 9.52 μg/g, Rc: 117.54 ± 10.48 μg/g, Rg2: 18.15 ± 4.67 μg/g, Rb2: 71.90 ± 13.79 μg/g, Rd: 272.38 ± 11.25 μg/g, and F2: 55.19 ± 2.91 μg/g, respectively). On the other hand, the water-soluble fraction (WFGE) showed relatively lower levels of these compounds. These differences are presumed to be attributable to the varying polarities of ginsenosides, which affect their solubility and distribution during fractionation. Li et al. [44] reported that the treatment with hot water of extracts of five-year-old ginseng roots, as well as their sequential fractions enriched in phenolic compounds, ameliorated UVB-induced photoaging in a mouse model. This effect was attributed to the regulation of key inflammatory and aging-related markers such as MMPs, COX-2, IL-1β, and IL-6. Similarly, Kwok et al. [45] demonstrated that ginsenoside Rb1 enhanced collagen production in human dermal fibroblasts stimulated with transforming growth factor-β1, significantly upregulating the expression of COL1A2, a collagen-associated protein. More recently, Choi et al. [46] found that treatment with ginseng root-derived exosome-like nanoparticles modulated reactive oxygen species (ROS) levels and improved the expression of apoptotic genes in UVB-irradiated and H_2_O_2_-treated human keratinocyte HaCaT cells. Additionally, they observed the suppression of skin aging and inflammation-related genes including MMP-2, MMP-3, MMP-9, COX-2, and IL-6, suggesting a protective role against UV and oxidative stress-induced skin damage. Taken together, these findings suggest that the anti-inflammatory and photoaging-protective effects observed in this study might be attributed to the biological activities of ginsenosides present in the KF-cultivated ginseng sprouts.

## 3. Materials and Methods

### 3.1. Preparation of Extracts and Crude Saponin Fractions

To prepare the KF used for cultivating *Panax ginseng* sprouts (GS), a fermentation protocol was adapted from a previously established method [20]. Briefly, a nutrient mixture containing 3% (*w*/*v*) defatted soybean extract (Hokyoung-Tech Co., Anseong, Republic of Korea), 2% (*w*/*v*) molasses, and 3% (*w*/*v*) desalted kelp powder was homogenized and heat-treated at 121 °C for 15 min. The mixture was then inoculated with *Saccharomyces cerevisiae* KCTC 17299 (Korea Collection for Type Cultures, Jeongup, Republic of Korea) at a final concentration of 0.01% (*v*/*v*) and incubated at 30 °C for 72 h in a shaking incubator (KoBiotech Co., Ltd., Incheon, Republic of Korea).

Following fermentation, the broth was pasteurized at 90 °C for 30 min, filtered through a 0.45 µm sterile membrane filter, and stored at 4 °C until use. To assess the biological activity and ginsenoside accumulation in ginseng sprouts cultivated with different nutrient sources, one-year-old *Panax ginseng* roots (Elounsesang Agricultural Corp., Yeonggwang, Republic of Korea) were washed thoroughly and soaked in a 25% (*v*/*v*) solution of KF and diluted with distilled water for 3 h [20]. The treated roots were then transplanted into a customized smart-farming chamber (MO Green Korea Co., Ltd., Gwangju, Republic of Korea), where they were grown under dark conditions at 25 ± 2 °C and 70 ± 5% relative humidity for 7 days. Then, they were exposed to light-emitting diode lamps for 21 days at a 142 μmol∙m-2∙s-1 photosynthetic photon flux density. After 28 days of cultivation, the ginseng sprouts were collected and stored at 4 °C prior to extraction procedures. For extraction, the ginseng sprouts were subjected to heat reflux extraction using 70% (*v*/*v*) ethanol at 85 °C for 3 h. The resulting solution was filtered and concentrated using a rotary vacuum evaporator (N-1200-B, Eyela, Tokyo, Japan).

Crude saponin fractions were then prepared with slight modifications to the procedure described by Shehzad et al. [47]. Specifically, 1 g of dried ginseng extract (FGE) was applied to an open column (15 cm × 3 cm inner diameter) packed with Diaion HP-20 resin (Sigma-Aldrich, St. Louis, MO, USA). Elution was performed sequentially using an ethanol–water gradient (0%, 20%, 70%, and 100% ethanol). Among them, the water fraction of crude saponin extract from FGE (WFGE) and the 70% ethanol fraction of crude saponin extract from FGE (EFGE) were subsequently evaporated, freeze-dried, and stored at −20 °C until further experiments.

### 3.2. Phytochemicals in Ginseng Sprouts

The TPC of ginseng sprout extracts and their crude saponin fractions was determined using a modified version of the Folin–Ciocalteu colorimetric assay, based on the procedure described by Ainsworth and Gillespie [48], with some modifications. In summary, the extract was treated with the Folin–Ciocalteu reagent, followed by neutralization using a sodium carbonate solution. The resulting mixture’s absorbance was measured at 760 nm using a spectrophotometer (Epoch2, BioTek Co., Winooski, VT, USA). Gallic acid (Sigma-Aldrich, ≥99% purity) served as the calibration standard, and TPC values were reported in micrograms of gallic acid equivalents per milligram of extract (μg GAE/mg).

To assess TFC, an aluminum chloride-based colorimetric technique was employed following the method described by Lyu et al. [49], with slight modifications. Briefly, 250 µL of the extract was combined with 100 µL of 1 M sodium nitrite and 1 mL of distilled water under dark conditions and allowed to stand for 5 min. Subsequently, 150 µL of 5% aluminum chloride was added, and the reaction was maintained in the dark for another 5 min. Thereafter, 500 µL of 10% sodium hydroxide and 500 µL of distilled water were added, followed by a final 10 min incubation in darkness. The absorbance was recorded at 510 nm using the spectrophotometer (Epoch2, BioTek). Quercetin (Sigma-Aldrich, ≥99% purity) was used as a reference compound, and TFC was expressed as micrograms of quercetin equivalents per milligram (μg QE/mg).

### 3.3. ABTS Radical Scavenging Assay

The antioxidant capacity of ginseng sprout extracts and crude saponin fractions was evaluated using the ABTS radical scavenging assay, as adapted from the method of Lyu et al. [50] with minor modifications. To generate ABTS radicals, 5 mL of a 7 mM ABTS solution was combined with 88 µL of 140 mM potassium persulfate. The resulting mixture was allowed to react in the dark at ambient temperature (23 ± 2 °C) for 16 h to ensure full radical formation. This solution was then diluted with distilled water in a 1:44 (*v/v*) ratio prior to use. For the assay, 100 µL of the diluted ABTS working solution was mixed with 100 µL of the sample extract in a 96-well microplate and incubated at room temperature for 5 min. The decrease in absorbance was recorded at 734 nm using the spectrophotometer (Epoch2, BioTek). The IC_50_ value, defined as the concentration required to inhibit 50% of the ABTS radicals, was calculated to represent antioxidant efficiency.

### 3.4. Cell Culture

Mouse macrophage RAW264.7 cells and human dermal fibroblast Hs68 cells were obtained from the American Type Culture Collection (ATCC; Rockville, MD, USA). Both cell lines were cultured in Dulbecco’s Modified Eagle Medium (DMEM; Cytiva, Marlborough, MA, USA) supplemented with 10% fetal bovine serum (FBS; Gibco, ThermoScientific Co., Waltham, MA, USA) and 100 U/mL penicillin-streptomycin (Gibco). The cells were maintained under standard culture conditions at 37 °C in a humidified incubator with 5% CO_2_. All experiments were performed using cells that were approximately 70~80% confluent to ensure optimal physiological conditions.

### 3.5. Cell Viability

The viability of RAW 264.7 and Hs68 cells was assessed using the Cell Counting Kit-8 (CCK-8; Dojindo Laboratories, Tokyo, Japan) according to the manufacturer’s instructions. Both cell types were seeded in 96-well plates at a density of 3 × 10^4^ cells/mL and 1 × 10^4^ cells/mL, respectively, with 100 μL of cell suspension added to each well. Subsequently, 100 μL of various ginseng sprout-derived samples including GE, FGE, WFGE, and EFGE were added to achieve final concentrations of 1, 3, 10, and 30 μg/mL. The cells were incubated for 24 h at 37 °C in a humidified atmosphere containing 5% CO_2_. Following the incubation period, 100 μL of the culture medium was gently replaced with fresh medium containing 10 μL of the CCK-8 reagent. After an additional incubation period, cell viability was evaluated by measuring the absorbance at 450 nm using a microplate reader.

### 3.6. Nitric Oxide Assay

The production of NO was indirectly measured by quantifying the accumulation of nitrite, a stable NO metabolite, using the Griess reagent [51]. RAW 264.7 macrophages were seeded into 96-well plates at a density of 5 × 10^4^ cells per well in 100 μL of complete DMEM and incubated overnight at 37 °C in a humidified atmosphere containing 5% CO_2_. To induce an inflammatory response, the cells were pre-treated with 1 μg/mL LPS for 1 h, followed by treatment with various concentrations of the GE, FGE, WFGE, and EFGE for 24 h. After treatment, 100 μL of culture supernatant from each well was transferred to a new 96-well plate, and 100 μL of freshly prepared Griess reagent composed of 0.1% N-1-naphthyl-ethylenediamine and 1% sulfanilamide in 5% phosphoric acid, mixed in a 1:1 ratio, was added. The mixture was incubated at room temperature for 10 min, and the absorbance was recorded at 550 nm using a microplate reader. Nitrite concentrations were calculated based on a standard curve generated with sodium nitrite.

### 3.7. Determination of Cytokine Levels

To assess the levels of pro-inflammatory cytokines, RAW 264.7 macrophages were seeded into 24-well plates at a density of 5 × 10^4^ cells per well and incubated overnight at 37 °C under 5% CO_2_. Cells were then stimulated with 1 μg/mL LPS in the presence or absence of varying concentrations of the GE, FGE, WFGE, and EFGE for 24 h. Following incubation, the culture media were collected for cytokine analysis. The concentrations of IL-1β and IL-6 in the supernatants were quantified using commercial enzyme-linked immunosorbent assay (ELISA) kits, following the manufacturer’s protocols. Briefly, 96-well ELISA plates were coated with capture antibodies diluted in coating buffer (100 μL per well) and incubated overnight at 4 °C. After washing, the plates were blocked with PBS containing 10% FBS. Diluted supernatants were then added and incubated at room temperature. Plates were subsequently washed and treated with biotin-labeled detection antibodies (100 μL per well), followed by HRP-conjugated secondary antibodies. A substrate solution was added for color development, and absorbance was measured using a microplate reader.

### 3.8. Measurement of Intracellular Procollagen

Hs68 human dermal fibroblasts were seeded into 6-well plates and incubated for 24 h under standard culture conditions. To induce photoaging, cells were exposed to UVB radiation at a dose of 30 mJ/cm^2^ using a UV Crosslinker BLX312 (Vilber Lourmat, Collégien, France). Following UVB exposure, the culture medium was replaced with phenol red-free DMEM supplemented with 1% fetal bovine serum (FBS), and the cells were treated with ginseng sprout extracts, including GE, FGE, WFGE, and EFGE, for 24 h. Intracellular procollagen levels were measured using a Procollagen Type I C-Peptide (PIP) enzyme immunoassay kit (Takara Bio Inc., Kusatsu, Shiga, Japan) in accordance with the manufacturer’s instructions. Test samples were diluted in a 1:5 ratio using the provided ELISA sample buffer. For each well, 20 μL of either the diluted sample or PIP standard solution was added, followed by the addition of 100 μL of antibody–POD conjugate. The plate was incubated at 37 °C for 3 h. Subsequently, 100 μL of substrate solution was added to each well and allowed to react for 15 min at 20 °C, after which 100 μL of stop solution was added to terminate the reaction. The absorbance was measured at 450 nm using a microplate reader.

### 3.9. Matrix Metalloproteinase-1 Inhibition Activity

Hs68 cells were seeded in 24-well plates and cultured for 24 h. The cells were then subjected to UVB irradiation and subsequently treated with GE, FGE, WFGE, and EFGE for an additional 24 h. Following treatment, the collagenase activity was assessed using a Human MMP-1 ELISA kit (R&D Systems Inc., Minneapolis, MN, USA) in accordance with the manufacturer’s guidelines. Absorbance was measured at 450 nm using a microplate reader. The level of MMP-1 was calculated and compared to the UVB-irradiated control group to evaluate inhibitory effects.

### 3.10. Western Blot Analysis

RAW 264.7 and Hs68 cells were harvested and washed three times with cold phosphate-buffered saline (PBS), followed by lysis using RIPA buffer (ThermoScientific Co., Waltham, MA, USA). Lysates were centrifuged at 15,000 rpm for 30 min at 4 °C, and the supernatants were collected. Protein concentrations were determined using the BCA Protein Assay Kit (Pierce Biotechnology, Rockford, IL, USA). Equal amounts of protein (20 μg) were mixed with 4× Laemmli buffer, denatured at 100 °C for 5 min, and resolved by 10% SDS-PAGE. Proteins were then transferred onto polyvinylidene fluoride membranes and blocked with 3% non-fat dry milk in TBS-T for 1 h at room temperature. Membranes were incubated overnight at 4 °C with primary antibodies specific to iNOS, COX-2, MMP-1, MMP-3, p38, phospho-p38, ERK, phospho-ERK, JNK, phospho-JNK, c-Fos, phospho-c-Fos, c-Jun, phospho-c-Jun, and β-actin (Santa Cruz Biotechnology, Santa Cruz, CA, USA). After washing, membranes were treated with HRP-conjugated goat anti-rabbit secondary antibodies. Protein bands were visualized using an enhanced chemiluminescence detection system and imaged using the ChemiDoc imaging system (Bio-Rad, Hercules, CA, USA). Band intensities were quantified using Image Lab software (Bio-Rad, Hercules, CA, USA, https://www.bio-rad.com/ja-jp/product/image-lab-software?ID=KRE6P5E8Z, accessed on 21 April 2025), and relative protein expression levels were normalized to those of the LPS-treated or UV-irradiated control groups as appropriate [52].

### 3.11. Identification and Quantification of Ginsenosides

Ginsenosides present in GE, FGE, WFGE, and EFGE were identified and quantified using HPLC-ELSD/MS. The analysis was carried out using an Agilent 1260 HPLC system equipped with an ELSD detector (G4260A) and an Agilent 6120 Quadrupole mass spectrometer with an electrospray ionization source. Chromatographic separation was performed on an Eclipse XDB C18 column (4.6 × 150 mm, 5 µm; Agilent Technologies, Santa Clara, CA, USA). The mobile phase consisted of solvent A (water) and solvent B (acetonitrile), both containing 0.1% formic acid. The gradient elution program was as follows: 20% B held for 5 min, increased to 35% B over 8 min (5–13 min), to 40% B over 10 min (13–23 min), then ramped to 85% B over 17 min (23–40 min), and held at 85% B for an additional 8 min. The flow rate was set at 0.6 mL/min, and the injection volume was 10 µL. Detection was performed at 203 nm. Quantification was achieved by constructing calibration curves based on the peak areas of known standard concentrations. Standard solutions were prepared using nine ginsenosides (Rg1, Rf, Rb1, Rc, Rg2, Rb2, Rd, F2, and Rg3; Sigma-Aldrich, purity >99%) as well as compound K (CK; Wuhan ChemFaces Biochemical Co., Ltd., Wuhan, China, purity >98%). Each standard was prepared in methanol at five different concentrations ranging from 10 to 200 µg/mL. Calibration curves were constructed, and limits of detection (LOD) and quantification (LOQ) were calculated using the formulas LOD = 3 × (SD)/S) and LOQ = 10 × (SD/S), where SD is the standard deviation of the response and S is the slope of the calibration curve.

### 3.12. Statistical Analysis

All experiments were conducted in triplicate, and the results are presented as mean values ± standard deviation (S.D.). Statistical analysis was performed using one-way analysis of variance (ANOVA) with SAS software version 9.4 (SAS Institute Inc., Cary, NC, USA). Differences between groups were assessed using Tukey’s multiple comparison test and Duncan’s multiple range test, with statistical significance set at *p* < 0.05.

## 4. Conclusions

In summary, this study demonstrates that *Panax ginseng* sprouts cultivated with kelp fermentates (KF) in a smart-farming system significantly enhance the accumulation of bioactive ginsenosides. Extracts and their saponin fractions exhibited potent antioxidant and anti-inflammatory properties by suppressing LPS-induced pro-inflammatory mediators in macrophages. Furthermore, they protected human dermal fibroblasts from UVB-induced photoaging by enhancing type I procollagen synthesis and inhibiting the expression of MMP-1 and MMP-3. Mechanistically, these effects were associated with the downregulation of the MAPK/AP-1 signaling pathway. These findings suggest that KF-cultivated ginseng sprouts could serve as an effective natural source for developing functional ingredients aimed at preventing skin inflammation and photoaging.

## Figures and Tables

**Table 1:** Nine ginsenosides and compound K (CK) contents in the ginseng sprout extracts and its crude saponins fractions from ginseng sprouts cultivated with kelp fermentates (KF).

Ginsenosides (μg/g)	GE	FGE	WFGE	EFGE
Rg1	57.16 ± 0.87 b	96.89 ± 1.64 c	1.60 ± 0.62 a	433.39 ± 32.07 d
Rf	N.D.	1.73 ± 0.62 a	N.D.	13.90 ± 2.59 b
Rb1	14.14 ± 14.14 b	19.21 ± 0.63 b	3.33 ± 0.25 a	68.48 ± 9.52 c
Rc	19.37 ± 0.81 b	32.09 ± 1.66 c	3.09 ± 0.22 a	117.54 ± 10.48 d
Rg2	2.86 ± 0.26 a	4.76 ± 0.56 a	N.D.	18.15 ± 4.67 b
Rb2	14.19 ± 0.91 a	19.21 ± 1.18 a	7.68 ± 0.38 a	71.91 ± 13.79 b
Rd	39.48 ± 1.76 b	79.41 ± 1.89 c	15.14 ± 0.81 a	272.38 ± 11.25 d
F2	2.27 ± 0.48 a	20.57 ± 0.81 b	3.33 ± 0.65 a	55.19 ± 2.89 c
Rg3	N.D.	N.D.	N.D.	N.D.
CK	N.D.	N.D.	N.D.	N.D.

Data are the mean ± S.D. of three independent measurements. Different letters show the differences in Duncan’s multiple range tests (*p* < 0.05) between values in the row. N.D., not detected; GE, ginseng extracts; FGE, the extract of KF-treated ginseng; WFGE, the water fraction of crude saponin extract from FGE; EFGE, the 70% ethanol fraction of crude saponin extract from FGE.

## Data Availability

Data are contained within the article.

## Appendix A

**Table A1:** Calibration curves, limits of detection (LOD), and limits of quantification (LOQ) for nine ginsenosides and compound K.

Ginsenosides	Retention Time (min)	Calibration Curve	Correlation Coefficient (r^2^)	Test-Range (μg/mL)	LOD (μg)	LOQ (μg)
Rg1	13.91	y = 6.1815x + 12.324	0.9996	10–200	1.13	11.34
Rf	18.38	y = 5.6109x + 12.12	0.9997	10–200	2.81	28.11
Rb1	18.94	y = 3.5427x + 5.6434	0.9999	10–200	2.39	23.90
Rc	19.81	y = 3.9614x + 7.4171	1.0000	10–200	6.82	68.22
Rg2	20.31	y = 6.3725x + 9.4992	0.9996	10–200	1.38	13.80
Rb2	20.75	y = 4.0161x − 0.5434	0.9994	10–200	1.86	18.56
Rd	23.24	y = 3.7723x + 4.7266	0.9993	10–200	3.04	30.40
F2	30.48	y = 4.6498x + 11.965	0.9996	10–200	1.56	15.57
Rg3	32.38	y = 5.7352x + 13.632	0.9999	10–200	N.D.	N.D.
CK	37.72	y = 6.5322x + 16.706	0.9996	10–200	N.D.	N.D.

Calibration curve: y equals peak area, and x equals concentration (μg/mL). Standard deviation (SD)/slope of the calibration curve (s) = 3; SD/s = 10. LOD, limit of detection. LOQ, limit of quantification. N.D., not detected.
